# Transactions of the Medical Association of Georgia

**Published:** 1885-01-20

**Authors:** 


### TRANSACTIONS OF THE MEDICAL ASSOCIATION OF GEORGIA.

The last meeting of this association took place at Macon, April 16, 1884, and was a meeting of more than usual interest. The copy of the Transactions, just handed in by the secretary, is neatly and elegantly gotten up, and is, we believe the largest copy issued by the society during the long years of its existence.

The table of contents is one of great variety and interest, and indicates the high order of talent and enterprise on the part of the profession in Georgia. We have only taken a cursory glance at the work, and have not time and space in this issue of our journal for a notice of all the papers of interest therein contained.

The address of the President, Dr. A. W. Calhoun, upon School Hygiene, in relation to its influence upon the vision of children, is one of ability and interest.

The report from the First Congressional District on Diseases of Women, by R. J. Nunn, is a paper of ability and of much practical interest, illustrated, and presented under separate heads, in a manner which evinces skill, care, and enterprise on the part of the writer.

The paper by Prof. Thomas S. Powell, on Gynæcology in the Fifth Congressional District, is one which evinces marked ability and thorough acquaintance with the subject of which he treats. The paper contains much useful information, and many points of practical interest to the profession. The Professor, though an advocate of specialism, remarks : "I would not be considered the advocate of a specialism which ignores the importance and necessity of a thorough knowledge of the principles and practice of medicine. It were well, in every case, that the party who proposes to devote himself to a specialty, especially in gynæcology, should first be thoroughly grounded in general principles, including medicine and all its collateral branches, and that he should for a time engage in active general practice before devoting himself to the specialty."

The paper of Prof. J. McF. Gaston, entitled "Explanation of the Pathology and Therapeutics of the Diseases of the Nerve centres, especially Epilepsy," has been favorably criticised by a number of journals. It evinces a thorough acquaintance with the physiological anatomy of the nervous system, and advances a plausable theory as explanatory of certain phases and purterbations, as well as of therapeutic impressions not satisfactorily accounted for by what are styled reflex manfestations. This property of the nervous system he pronounces the "*excito-dynamic element*." To this power, rather than by the slow process of absorption, he attributes the almost instantaneous action of certain medicinal agents when injected beneath the cuticle. So "the notable effects of dry cups, sinapisms, etc., upon the surface are due to the intimate relations established through this channel of communication with the internal parts of the body." "It is this element of nerve power which suffers detriment in the vital failure called shock from severe injuries, and there must be a partial paralysis of that property of the nervous system to which I have applied the term excito-dynamic."

For want of space we must defer now to notice the interesting Report of Special Cases by Professor Nicloson; Antisepsis in Ovariotomy, etc., by Dr. Robert Battey; Jequirity—its uses in the treatment of granular lids, etc., by Prof. A. G. Hobbs; Syphilis as a Sociological Problem, by Dr. Eugene Foster; and other papers by the following gentlemen, to wit: T. F. Walker, M. D.; T. M. Holmes, M. D.; W. B. Wells, M. D.; James M. Hull, M. D.; J. P. Stevens, M. D.: W. C. Gibson, M. D.; H. J. Williamson, M. D.; C. W. Hickman, M. D.; H. McHatton, M. D.; W. O. Daniel, M. D., A. F. Akland, M. D.; K. P. Moore, M. D., J. W. Flanders, M. D.; N. G. Gewinner, M. D.; T. L. Lallerstedt, M. D.; W. B. Parks, M. D.; S. D. Brantley, M. D.; and N. P. Jelks, M. D.

The next meeting of the Association is appointed for Savannah on the third Wednesday in April, 1885. W.

